# The Effect of Temperature on the Inflorescence Formation Model for *Phalaenopsis*

**DOI:** 10.3390/plants13091280

**Published:** 2024-05-06

**Authors:** Jiunyuan Chen, Chiachung Chen

**Affiliations:** 1 Africa Industrial Research Center, National Chung Hsing University, 250 Kuokuang Road, Taichung 40227, Taiwan; kepler0953@gmail.com; 2 Department of Bio-Industrial Mechatronics Engineering, National Chung Hsing University, 250 Kuokuang Road, Taichung 40227, Taiwan

**Keywords:** regression analysis, *Phalaenopsis*, inflorescence formation model, accumulation temperature

## Abstract

*Phalaenopsis* orchids are a popular ornamental plant in the flower market. During some festivals, demand increases significantly. These mature orchids must be placed in cooling rooms for inflorescence formation at specific times to increase the financial return from their sale. The purpose of this study is to evaluate the effect of day and night temperatures on the inflorescence formation percentage using the proposed sigmoid model. Four varieties that are cultured in different vegetative temperature regimes are placed in a cooling room. An empirical inflorescence formation model is proposed as a management tool to predict the inflorescence formation percentage for *Phalaenopsis.* Some data sets from previous studies are used for comparison. The accumulation temperature is calculated using the day and night temperatures and is an index to predict the inflorescence formation percentage. The results show that there is a similar distribution of the inflorescence formation percentage and accumulation temperature for the four varieties. The proposed sigmoid model has a good fitting ability for the inflorescence formation percentage. This inflorescence formation model from the pooled data sets allows quantitative microclimate management of the vegetative and cooling room.

## 1. Introduction

*Phalaenopsis* is a popular ornamental plant in the flower market. Many colors and sizes of flowers and the lengthy flowering duration make growing these orchids a very profitable enterprise. During special festivals, such as New Year, Valentine’s Day, and Mother’s Day, demand increases significantly so the timing of flowering for the orchids significantly affects the profitability of an enterprise that grows them [1,2].

These orchids are cultivated in vegetative rooms maintained at high temperatures for growing and placed in cooling rooms for inflorescence formation and flowering [3,4,5]. To provide potted *Phalaenopsis* to the market at the appropriate time, inflorescence formation and stem elongation must be controlled by the producer. The environmental factors that affect inflorescence formation include day and night temperatures and light intensity [1,2,3,4]. The definition of flowering for *Phalaenopsis* is the opening of the first floret.

A cooling room in a greenhouse is used by commercial growers of *Phalaenopsis*. Environmental factors such as light intensity, photoperiods, day temperature, and night temperature are controlled automatically. Light intensity and photoperiods are tightly controlled in cooling rooms for *Phalaenopsis* [1,4]. A mathematical equation that expresses the relationship between the inflorescence formation characteristics and environmental factors is very useful for management.

Thomas [6] studied the effect of temperature on flowering. The relationship between verbalization and the initiation of a lower temperature is explained using physiological theory. A lot of information was introduced about the factors affecting the flowering of plants. The relationship between low temperature and flower initiation is controlled by a balance between inhibitors and initiation. Higher temperature enhances the inhibitors and lower temperature induces the initiation [6]. Khodorova and Boitel-Conti [7] proposed the relationship between the cold environment, auxin and gibberellin interactions, and the growth response such as water status, respiration, and carbohydrate distribution to explain the role of temperature in the flowering. The lower temperature is required to optimize the flower meristem of differentiation. Xuan et al. [8] explained the temperature-dependent regulation of flowering at the molecular level. The low temperature induces the splicing of the transcription factors of flowering locus M (FCM). However, no quantitative relationship was proposed in the previous study. 

Lopez and Runkle [9] proposed two linear equations to describe the effect of temperature on the time of flowering and the rate of progress to flowering for hybrid *Zygopetalum* Redvale orchids. Lopez and Runkle [10] regulated the temperature and photoperiod for the flowering of *Miltoniopsis* orchids but no equations were established. Blanchard and Runk [11] noted that only the day temperature controls the flowering of the *Phalaenopsis* and the day/night temperature need not differ. In terms of the prohibited technique of inflorescence of *Phalaenopsis* orchids, Newton and Runlcle [12] proposed that a temperature greater than 29 °C for 8 h or 12 h is necessary for inflorescence prohibiting. 

Lin et al. [13] developed a method to defer the flowering of noble dendrobium orchids by placing orchids in a low-temperature environment. Two linear equations were derived to determine the effect of maintaining a low temperature for specific weeks on the time of flowering. Paradiso et al. [14] used three day/night temperature regimes (21/19 °C, 23/21 °C, and 19/17 °C) to determine the relationship between the inflorescence formation percentage and the time from the beginning of cooling but only the coefficient of determination is reported for three data sets. 

Paradiso and De Pascale [15] determined the effect of plant size, temperature, and light intensity on the flowering performance of *Phalaenopsis* and showed that temperature regimes do not significantly affect the flowering time. Only two day/night temperature regimes (21/19 °C and 19/17 °C) were used for comparison. 

The mathematical model is very useful for managing orchid production. It provides strict information to quantify the factors affecting the growth and flowering of orchids. Jin et al. [16] established a plastochron index model for four commercial cultivars of *Phalaenopsis* based on a 20-mm reference leaf length. Logarithmical transformed leaf lengths versus time were proposed with a linear regression analysis used to evaluate three criteria of the plastochron index. Their results indicated that fulfilled leaves fitted all three criteria for the plastochron index. However, the plastochron index cannot be used for the slow-growing *Phalaenopsis*. There were significant differences between the leaf growth curves for the four varieties. The plastochron index cannot fully express the growth characteristics of *Phalaenopsis*.

Chen et al. [17] studied the factors affecting the growth characteristics of *Phalaenopsis* leaves and measured the leaf length of two *Phalaenopsis* varieties under different environmental conditions such as daily temperature, light intensity, and fertilizer concentrations to establish growth characteristics. The consistency of the fitting agreement of the three nonlinear growth equations was compared using the leaf length data of the two varieties. The results showed that the logistic equation was the best equation to describe the relationship between leaf length and culture days. Environmental conditions and fertilization rates significantly affected the leaf length of both cultivars. The growth rate of a variety is significantly affected by daytime temperature and light intensity. However, the growth rate of the other species was not affected. The author proposed that by fitting nonlinear growth equations to plant growth data and analyzing its parameters, they can be used to study factors affecting the growth characteristics of *Phalaenopsis* leaves [17].

Chang et al. [18] developed a simple and nondestructive technique to estimate the new roots of potted *Phalaenopsis*. A simple and nondestructive technique, based on the line intersect principle, was developed for simultaneously estimating more traits of the newly grown roots of potted *Phalaenopsis* plants. Two kinds of root distribution counting pots and large and small drawing grids were used to determine the grid number of the newly grown roots of each plant. The linear regression equation was used to analyze the relationship between the number, fresh weight, dry weight, and length of the roots and these counting numbers. However, the coefficients of determination of these equations were lower and the mean squared errors were higher. The root length equation had a prediction ability [18].

Chen [19] evaluated the effect of temperature on flower stem elongation and established a model using statistical techniques. The cultivation temperature was used as the independent variable. The flower stem length of different varieties was measured under different temperature conditions. The growth model is a Logistic equation and its three parameters are the maximum flower stem length, growth rate, and the inflection point of the maximum growth rate. Categorical variables were used to assess the effect of variety on these three parameters. The results indicate that stem growth rate is affected only by daytime temperature. Maximum flower stem length is affected by daytime temperature (negative effect) and night temperature difference (positive effect) [19]. The findings could provide a practical method to regulate flower stem elongation conditions by adjusting temperature settings.

Cave et al. [20] proposed a multiplicative model to describe the development rate for *Brunosis australis*, which is affected by temperature and the photoperiod. The temperature function uses the base, optimum, and maximum temperature.

To enhance the smart production of orchids, Chen et al. [21] study the shape characteristics and the allometry of *Phalaenopsis* leaves. The allometry of *Phalaenopsis* leaves of four varieties was investigated. The leaf development and the total leaf area models were developed. Two models, the top and bottom models, were used to calculate the total leaf areas. No significant differences were found in the length ratio, width ratio, and area ratio of consecutive leaves of the same varieties. However, there are significant differences in these leaf characteristics between varieties. The top model has better predictive power than the bottom model. The accuracy of total leaf area measurement can be ensured by measuring more than 30 samples [21]. The total leaf area model of this study can be incorporated into decision-making models for intelligent management.

This study uses temperature as the factor that has a dominant effect on inflorescence formation. The effect of the day and night temperatures on the inflorescence formation percentage is determined. Four varieties that were cultured in different temperature regimes were placed in a cooling room. The inflorescence formation percentages are used to verify the inflorescence formation percentage model. Data from previous studies are compared with the results of this study. 

## 2. Results

### 2.1. The Inflorescence Formation Percentage for Four Varieties of Phalaenopsis

**Figure 1:**
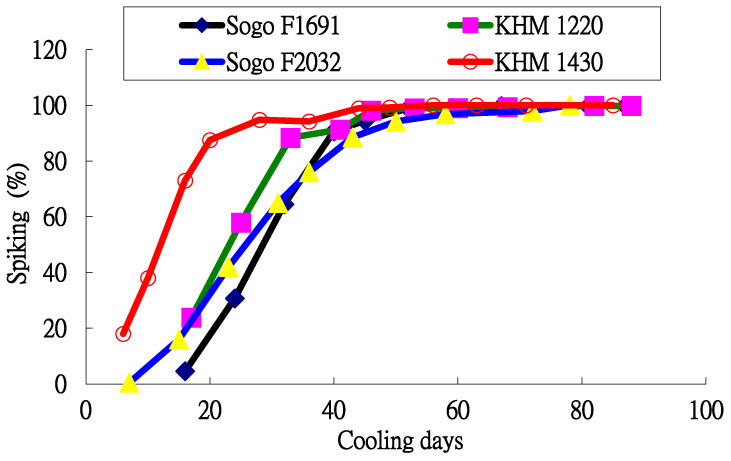
Relationship between inflorescence formation percentage and cooling time for four flower varieties.

The relationship between the inflorescence formation percentage and the number of cooling days for four *Phalaenopsis* varieties is shown in Figure 1. The inflorescence formation percentage for *Phalaenopsis* plants increases as the length of the cooling period increases but the distribution curves for the four varieties are not the same. 

**Figure 2:**
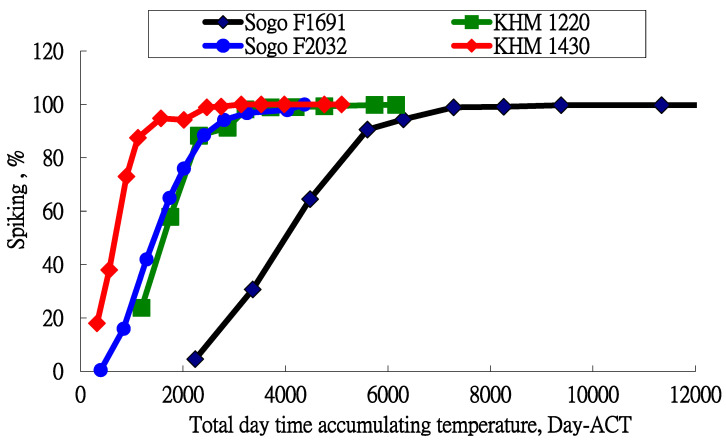
Relationship between inflorescence formation percentage and daytime accumulated temperature for the four varieties only.

If only the accumulation of the day temperature is considered, the Day-ACT value is calculated using Equation (6). The relationship between the inflorescence formation percentage and the accumulation temperature (°C-hour) of day, Day-ACT, is shown in Figure 2. Two varieties, KHM1220 and Sogo F2032, have similar distribution curves but the varieties, KHM 1430 and Sogo F1691, have different distribution curves. These curves were inconsistent if only the cooling effect during the day was considered.

**Figure 3:**
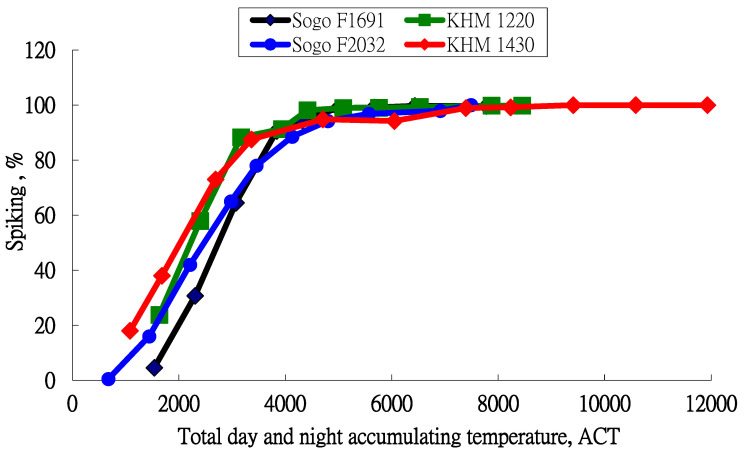
Relationship between inflorescence formation percentage and accumulated temperature for day and night for the four varieties.

The distribution of the inflorescence formation percentage and the accumulation temperature that is calculated using Equation (5) are shown in Figure 3. These four curves have a similar distribution. In terms of the effect of the cooling temperature on the initiation of inflorescence formation, day and night temperatures both have a significant effect so the calculation of the accumulation temperature using the day and night temperatures is an index to predict the inflorescence formation percentage.

Using the data in Figure 3, the inflorescence formation equation is calculated by regression analysis. 

There is a higher R^2^ and a smaller s value for the following empirical equation:

(1) $$Y=\frac{99.278}{1+38.585\;Exp(-ACT/662.397)},\;R^{2}\;=\;0.9863,\;s\;=\;3.623$$

### 2.2. Inflorescence Formation Data for Previous Studies 

**Figure 4:**
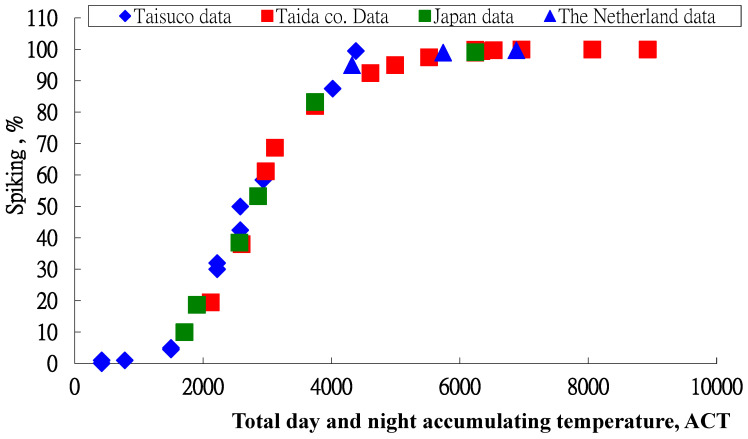
Relationship between inflorescence formation percentage and accumulated temperature for day and night for data from previous studies.

Four data sets from previous studies are used to validate this model. All data sets are shown in Figure 4. 

The consistent distribution of these data sets shows that the inflorescence formation percentage for *Phalaenopsis* can be expressed as a function of day and night accumulation temperatures (°C-hours). The regression equation for these data sets is

(2) $$Y=\frac{98.997}{1+169.87\;Exp(-ACT/534.504)},\;R^{2}\;=\;0.9852,\;s\;=\;3.835$$

### 2.3. The Pool of Data Sets

**Figure 5:**
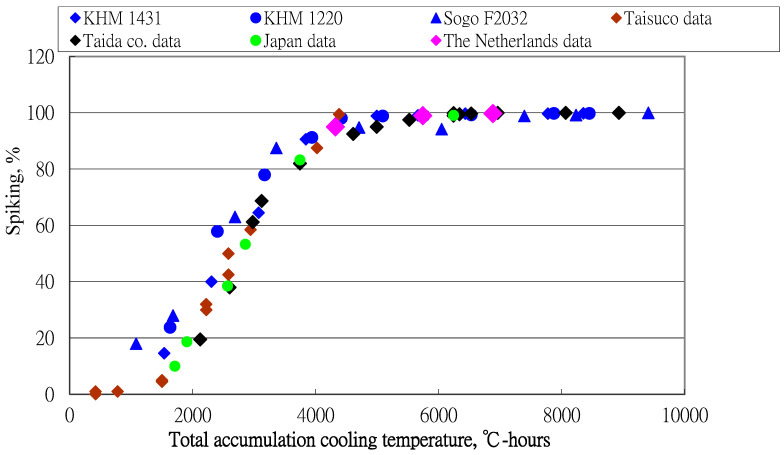
Relationship between inflorescence formation percentage and accumulated temperature for day and night data for this study and previous studies.

Figure 5 shows the data distribution for the data sets for this study and the previous studies. All data are pooled and the empirical equation is written as

(3) $$Y=\frac{99.3118}{1+83.4511\;Exp(-ACT/594.1813)},\;R^{2}\;=\;0.9826,\;s\;=\;4.865$$

This inflorescence formation model can be used as a management tool to predict the inflorescence formation percentage for *Phalaenopsis.*

## 3. Discussion 

The timing of blooming for potted *Phalaenopsis* is controlled by the inflorescence formation and the elongation of the stem. The flower stem elongation model was proposed and validated by Chen [19]. The result of this study shows that two models can be integrated to create a smart management system for the orchid industry. The temperature is adjusted using the inflorescence formation and flower stem elongation models.

Thomas [6] proposed the physiology of flowering and noted that temperature affects the chemical reaction rate of a plant. The study of its effect on flowering must be based on this process. The quantitative effect of low temperature is described as the initiation effect. This study establishes an inflorescence formation model for a quantitative effect.

The induction of flowers has been the subject of studies. Newton and Runkle [12] noted that higher temperatures inhibit the inflorescence formation of *Phalaenopsis*. Jeong et al. [22] reported that a temperature of more than 28 °C delays the initiation of inflorescence formation for *Phalaenopsis* Queen beer ‘Mantefon’. For higher temperatures, there is no accumulation temperature so the inflorescence formation equation is used reasonably to describe the degree to which high temperatures inhibit the initiation of *Phalaenopsis*. 

Blanchard and Rankle [11] showed that only the daytime temperature controls the flowering of *Phalaenopsis*. This study by Blanchard and Rankle [11] used only two varieties. In this study, the accumulation curves use only the daytime temperature, which is shown in Figure 2. Two varieties have a similar distribution and the others do not. This is explained by the abundance of varieties. Different *Phalaenopsis* varieties have different genetic characteristics. The results of this study show that day and night temperatures both control the inflorescence formation percentage. The daytime temperature alone cannot be used as the only factor that affects the initiation of inflorescence for all *Phalaenopsis* varieties.

For a study of the effect of the day/night temperature on flower induction by Paradiso et al. [14], only three thermal regimes were used: RT(21/19 °C), H(23/21 °C), and LT(19/17 °C). The results of the study show that the distribution of the relationship between the inflorescence formation percentage and the number of cooling days for the three temperature regimes is significantly different. This result shows that day and night temperatures both have a significant effect on inflorescence formation initiation. 

A study of the factors that affect the flowering of *Phalaenopsis* by Paradiso and De Pascale [15] showed that the sensitivity to induction temperature for *Phalaenopsis* is affected by the thermal past: the temperature during the vegetative period. In this study, these are denoted as T_vd_ and T_vn_ in Equation (2). It indicated that the inflorescence formation model developed in this study can be used to illustrate the results of this previous study.

To study the energy-saving method of controlling night temperature for vegetative growth of *Phalaenopsis*, Pollet et al. [23] experimented with eight varieties during complete vegetative cultivation in a greenhouse with day/night temperature set points of 28/28 °C, 29/23 °C, and 29/17 °C for 30 weeks. During week 25, fluorescence initiation increased rapidly for the 29/23 °C treatment compared to the 28/28 °C treatment. The results could explain the difference between the accumulation temperatures at cooling night temperatures. 

Zhang et al. [24] studied the effect of low-temperature accumulation on the flowering of *Prunus mune* and showed that the flowering rate and bud development in the 6 °C treatment were better than those in the 10 °C treatment. Noack et al. [25] observed the effect of two low-temperature treatments of four durations on the flowering of three cultivars of *Hebe* Comm ex. Juss. The low-temperature accumulation has a significant effect on the flowering characteristics. These authors proposed a qualitative description of the effect of low-temperature accumulation on the flowering. However, no quantitative equations were proposed. 

In the cultivation guide of *Phalaenopsis* used in The Netherlands [1,3,4], there are three layouts in the orchid greenhouse, vegetative growth, inflorescence formation, and flowering. The day and night temperatures are controlled strictly and fixed.; so, the orchids could be supplied according to the pre-planned schedule. However, the energy cost is high. In this study, the inflorescence formation percentage of *Phalaenopsis* could be predicted with the temperature accumulation model. It provides a useful tool to help the management of orchid production. 

Computers are used to control the microclimate so the day and night temperatures in vegetative and cooling rooms where *Phalaenopsis* is grown are controlled and recorded. A flower stem elongation model was developed by Chen [16] and an inflorescence formation model developed in this study can be used to describe the relationship between the inflorescence formation percentage and the accumulation temperature. Both models can be used to manage the temperature of cultivation areas by orchid companies. 

Digital management of greenhouse production has become the mainstream development of the floral industry. Modern greenhouses have many temperature, light, and other sensors. The greenhouse is equipped with various pieces of heating and cooling equipment, which can adjust the internal temperature precisely. The temperature accumulation values inside the greenhouse can be calculated and adjusted by these sensors, control equipment, and microcomputer systems, so the quality and timing of the market supply of flower products can be strictly controlled. Establishing a model of low-temperature accumulated value, combined with the microcomputer software system, is the basis of smart production. Therefore, the study of the relationship model between the cumulative low-temperature value of orchids and the inflorescence formation percentage has an innovative value.

## 4. Materials and Methods

### 4.1. Test Materials

The inflorescence formation experiment was conducted using a cooling room in a greenhouse in Datsun, Chung-Hwa, Taiwan. The cooling temperature was maintained at 22 °C during the day and 19 °C at night. The light intensity was at 220 μmolm^−2^s^−1^ and was controlled using artificial light and shading nets. The light period was maintained at 14 h.

Four commercial varieties (KHM1431, KHM1220, Sogo F1691, and Sogo F2032) were used to determine the effect of temperature on inflorescence formation. One thousand sample plants for each variety were used. The plants were cultivated at different orchid nurseries during the vegetative stage. All of the potted *Phalaenopsis* for this study were 18-month-old plants that had been cultivated in 10.5 cm transparent plastic pots with all sphagnum moss. During the inflorescence formation period, these plants were irrigated regularly using clean water with reverse osmosis and 10N-30P-20K liquid fertilizer (Hyponex Corp., Marysville, OH, USA). 

The characteristics of the four commercial varieties are listed in Table 1.

The plants were placed in a cooling room and the inflorescence formation condition was observed visually in terms of the appearance of the floral buds and the inflorescence formation percentage was calculated. 

### 4.2. Model Development

*Phalaenopsis* orchids must endure a period of low temperature to induce stemming. The day and night temperature and the duration of the low-temperature regime significantly affect the rate of inflorescence formation and the time that is required for inflorescence formation. The model that is proposed by this study is called an accumulation temperature model. A form of the sigmoid equation is used, as follows:

(4) $$Y=\frac{Ymax}{1+B\;Exp(-ACT/K)}$$

where Y is the inflorescence formation percentage, $Y_{\max}$ is the maximum inflorescence formation percentage for a specific variety, and B and K are parameters and ACT is the accumulated cooling temperature.

ACT is calculated as:

ACT = Σ (T_vd_ − T_cd_) H_d_ + Σ (T_vn_ − T_cn_) H_n_ (5)

where T_vd_ is the day temperature during the vegetative stage, T_cd_ is the day temperature in the cooling room for inflorescence formation, T_vn_ is the night temperature during the vegetative stage, T_cn_ is the night temperature in the cooling room for inflorescence formation, H_d_ is the length of day (hours) during the initial stage in the cooling room, and H_n_ is the length of night (hours) during the initial stage in the cooling room. 

If only the cooling temperature during the day is considered, the accumulated cooling temperature during the day is Day-ACT. Day-ACT is calculated as

Day-ACT = Σ (T_vd_ − T_cd_)H_d_ (6)

### 4.3. Literature Data

Four data sets from previous studies are used.

- Taiwan Sugar Corporation Research Report, 15 data sets [26];
- The experimental report of Taida Co., 15 data sets [27];
- Japanese research, 6 data sets [28];
- The study by Anthura b.v. in the Netherlands, 3 data sets [1,3].

### 4.4. Statistical Analysis

Data analysis used Sigmaplot 13.0 (SPSS Inc., Chicago, IL, USA). The coefficient of determination *R*^2^ and the estimated standard errors *s* are used to determine the fitting of the nonlinear equation.

## Figures and Tables

**Table 1:** Plant and flower characteristics for the four *Phalaenopsis* orchid varieties for the experiment.

	I-Hsin KHM 1431	I-Hsin KHM 1220	Sogo F1691	Sogo F2032
Final plant height (cm)	70	70	50	30
Flower size (cm)	10.5	11.0	8.5	4.5
Flower color	White	Red	Yellow	Pink
Vegetative temp. (°C)	32/24 °C	27/23 °C	26/23 °C	26/23 °C

## Data Availability

Data are contained within the article.
